# 嵌合抗原受体T细胞治疗相关长期血细胞减少

**DOI:** 10.3760/cma.j.issn.0253-2727.2023.10.018

**Published:** 2023-10

**Authors:** 敖 李, 茹 冯

**Affiliations:** 南方医科大学南方医院血液内科，广州 510515 Department of Hematology, Nanfang Hospital, Southern Medical University, Guangzhou 510515, China

嵌合抗原受体T细胞（CAR-T细胞）免疫疗法是血液系统恶性肿瘤的一种新型治疗策略，在复发/难治弥漫大B细胞淋巴瘤（DLBCL）、多发性骨髓瘤、急性淋巴细胞白血病（ALL）等恶性血液病患者中已取得显著的疗效。CAR-T细胞为一种有活性的免疫细胞药物，在治疗过程中会引发许多独特的毒性作用，如细胞因子释放综合征（CRS）、免疫效应细胞相关神经毒性综合征（ICANS）、噬血细胞性淋巴组织细胞增生症（HLH）等。血液学毒性是CAR-T细胞治疗常见的不良反应之一，输注30 d后血细胞计数持续低下提示预后不良，临床上应高度重视。本综述关注CAR-T细胞治疗后长期血细胞减少的影响因素及处理对策，旨在探讨早期识别高危患者，预防相关不良事件发生，并及时为患者提供有效的治疗。

一、概述

CAR-T细胞治疗通常用于常规疗法如化疗、放疗及造血干细胞移植（HSCT）[1]疗效不佳的恶性血液病患者。目前，CAR-T细胞治疗的常用的靶点有CD19、CD22、CD38、B细胞成熟蛋白（BCMA）、CD123、CD33、CD30、G蛋白偶联受体C5家族亚型D（GPRC5D）等。

血细胞减少是大多数CAR-T细胞治疗后常见的不良反应。CAR-T细胞输注后早期出现的血细胞减少与清除淋巴细胞的预处理方案相关。然而，有研究发现未接受清除淋巴细胞预处理方案的患者可出现血细胞减少的情况，表明CAR-T细胞存在直接造成骨髓抑制的机制[2]–[5]。血细胞计数下降持续到CAR-T细胞输注30 d后依然无法恢复，提示这种现象不能单独用化疗毒性来解释，大量的研究中也未发现预处理与其显著相关。长期血细胞减少将影响CAR-T细胞治疗的疗效及生存质量。参考文献[6]–[7]，本综述将CAR-T细胞治疗后长期血细胞减少定义为CAR-T细胞输注后超过30 d的≥3级的血细胞减少（ANC<1.0×10^9^/L或HGB<80 g/L或PLT<50.0×10^9^/L）。

二、CAR-T细胞治疗后长期血细胞减少的影响因素

（一）不同的CAR结构

不同CAR-T产品的区别主要在于治疗靶点和共刺激结构域（costimulatory domain）的不同。常用的靶点有：靶向B细胞的CD19、CD22，靶向骨髓瘤细胞的BCMA和GPRC5D等。除靶点外，共刺激结构域也赋予了CAR-T产品不同的特性。CAR-T细胞中的共刺激结构域可实现协同刺激分子和细胞内信号的双重活化，使T细胞持续增殖并释放细胞因子，提高T细胞的抗肿瘤能力。

不同类型的CAR-T细胞治疗后，3～4级血细胞减少的发生率及恢复率不同。如针对B细胞淋巴瘤、ALL的两项研究ZUMA-1[8]（axic-cel，靶向CD19，共刺激结构域CD28）及JULIET[9]（tisa-cel，靶向CD19，共刺激结构域4-1BB）中，ANC减少、HGB减少、血小板减少的发生率分别为78％、43％、33％及34％、39％、28％；而在针对多发性骨髓瘤的研究CARTITUDE-1[10]（cilta-cel，靶向BCMA）及OriCAR-017[11]（靶向GPRC5D）中，相应的发生率为95％、68％、60％及100％、70％、90％。这种不良反应的恢复率也有差异。Jain等[12]分析了18岁以上接受不同CAR-T（30例接受axic-cel治疗；10例接受tisa-cel治疗；37例接受CD19-28z CAR-T治疗；6例接受BCMA CAR-T治疗）治疗的淋巴瘤、ALL及多发性骨髓瘤患者的临床数据，结果显示CAR结构与血细胞恢复率间具有相关性：输注后1个月，24％的患者达到全血细胞计数完全恢复，其中axic-cel、tisa-cel、CD19-28z CAR-T及抗BCMA CAR-T组恢复率分别为17％、70％、19％、17％（*P*＝0.008）；输注后3个月，相应的恢复率为42％、100％、28％、50％（*P*＝0.050）。

以上研究表明，不同的CAR结构（治疗靶点及共刺激结构域）存在的内在差异可引起治疗后不同程度的血液学毒性。在靶向CD19的治疗中，以4-1BB为共刺激结构域相较于CD28而言血液学毒性更轻，≥3级血细胞减少发生率更低；且接受4-1BB CAR-T细胞治疗的患者能更早恢复，长期血细胞减少的发生率更低。

根据目前研究，CAR-T血液学毒性差异发生的可能机制为：持续暴露于抗原和与抗原无关的信号转导可引起CAR-T细胞的耗竭，在此过程中，细胞表达高水平的抑制性受体（如PD-1、TIM-3、LAG-3）以及衰竭相关转录因子[13]，而以4-1BB为共刺激结构域的CAR-T细胞与CD28相比，衰竭相关分子的表达减少，且细胞表面的免疫突触分布更均匀，能有效缓解T细胞的耗竭[14]。受到抗原刺激后，4-1BB CAR-T细胞向中央记忆T细胞分化，而CD28 CAR-T细胞则向效应记忆T细胞分化；4-1BB CAR-T细胞在体内扩增的速度较慢，但峰值更高、效应功能更强、可持续表达的时间更长，其缓慢扩增的特性造成的不良反应程度也更轻[15]。此外，部分CAR-T细胞的靶点为白血病细胞与正常造血干/祖细胞中共同表达的抗原（如CD123、CD33），可对造血细胞造成直接损伤，发生长期的骨髓抑制[16]–[18]。

（二）治疗过程中相关因素

1. 既往治疗及桥接治疗：在一项1b/2期临床试验[19]中，共有29例接受CD19-28z CAR-T细胞输注的复发/难治B-ALL或非霍奇金淋巴瘤的儿童及成人患者纳入分析。结果显示，在接受CAR-T细胞治疗前1年内进行过HSCT的患者中有5例（5/6）存在长期血细胞减少；在治疗前>1年行HSCT或无HSCT史患者中为5例（5/23）。研究提示输注CAR-T前1年内的HSCT治疗是发生长期血细胞减少的危险因素（*P*＝0.010）。在另一项研究[7]中，既往接受≥3线治疗也与长期血液学毒性之间存在统计学意义（*P*＝0.040）。以上研究提示，既往抗肿瘤治疗方案强度较高/治疗线数较多的群体，其总体治疗强度可能反映了患者较高的肿瘤负荷，以及多线/高强度治疗造成的造血储备功能不足，可能加剧CAR-T细胞治疗后长期持续的骨髓抑制状态。

桥接治疗（BT）定义为采集外周血单个核细胞和清淋巴细胞预处理之间进行的抗肿瘤治疗，可分为无桥接治疗、单用皮质类固醇激素、化疗、放疗以及联合治疗，其中化疗又可分为高剂量化疗（HDT）、低剂量化疗（LDT）及利妥昔单抗联合苯达莫司汀和维泊妥珠单抗治疗（RBP-BT）等。Claire等[20]分析了375例接受CD19 CAR-T治疗的复发/难治大B细胞淋巴瘤患者数据，其中87％（326/375）接受了不同类型的桥接治疗。在CAR-T输注后1个月时，≥3级的血小板减少发生率与BT类型之间存在统计学意义（*P*<0.001），其中接受化疗的患者发生率最高（56.0％），无BT组发生率为20.6％。与无BT组相比，BT组的患者≥3级的中性粒细胞减少发生率更高［（40％～45％）对27.3％；*P*＝0.051］，其中在接受化疗的患者中，HDT组的发生率最高（HDT对LDT，*P*＝0.012及HDT对RBP-BT，*P*＝0.002）。由于在选择桥接治疗方案时，常选择具有高风险疾病特征［如多淋巴结外侵犯、美国东部肿瘤协作组（ECOG）评分为1、高LDH、晚期或疾病快速进展等］的患者进行HDT，可能造成结果结果偏倚，研究具有一定的局限性。

2. 基线期造血储备能力：基线（定义为清淋巴细胞预处理前的时间点）骨髓内肿瘤负荷已被证实是中性粒细胞延迟恢复的独立因素[21]。基线血细胞减少可能反映了骨髓储备能力不足，研究表明其（尤其是血小板）与CAR-T细胞治疗后血细胞减少的发生和持续时间呈负相关（*r*＝−0.43, *P*＝0.001）[22]。基线期绝对淋巴计数低提示血液中的免疫抑制成分较少，在有利于CAR-T细胞治疗疾病的同时也可由于免疫炎症介质升高导致造血干细胞功能受损[23]。

3. CRS/ICANS及炎症标志物/细胞因子水平：CRS和ICANS是CAR-T输注后常见的不良反应。10％～30％的患者在输注CAR-T后可发生≥3级的CRS[24]，常于输注后1～14 d发病，持续时间通常为1～10 d。ICANS可与CRS同时或先后发生。研究证明CRS与长期血细胞减少之间存在较强相关性[21],[25]。Jain等[12]分析了41例（35例接受CD19-28z CAR-T治疗；6例接受抗BCMA CAR-T治疗）患者接受治疗1个月时的血细胞计数等临床数据，结果表示≥3级的CRS/ICANS与输注1个月时血细胞计数恢复之间呈负相关。发生4级CRS的患者出现血细胞减少的速度更快，谷值更低，恢复所需的时间也更长[19]。除CRS级别外，CRS发生时间、达峰时间、持续时长及托珠单抗/糖皮质激素的使用也与之相关[21],[26]。

CRS由大量细胞因子释放产生的炎症风暴引起。虽然在Jain等[12]及Rejeski等[22]的两项研究中均未发现治疗过程中与3～4级CRS相关的血清白细胞介素-6（IL-6）同长期血细胞减少之间的联系，但Juluri等[27]发现，血清IL-6峰浓度越高，血细胞减少的持续时间越长，TGF-β则相反。其他许多炎症因子的变化也与之相关，如血清基质细胞衍生因子-1（SDF-1）、IL-10、D-二聚体、CRP、铁蛋白等峰值[19],[21],[28]–[29]。

高水平细胞因子可对骨髓造成炎症损伤[28],[30]，促使炎症性贫血的发生，其中可能存在的机制为：①在炎症条件下，TNF-α可诱导粒细胞祖细胞/前体细胞中CD40显著上调，与细胞表面本身低水平表达的CD40L交联作用，通过Fas途径促进粒细胞祖细胞/前体细胞的凋亡，抑制粒细胞集落生长[31]。②早期B细胞发育依赖于SDF-1[32]。在CAR-T细胞治疗后B细胞恢复的过程中，骨髓微环境中SDF-1持续表达，促使造血干细胞（HSC）向B细胞谱系分化，影响中性粒细胞从骨髓排出，导致了其计数的延迟恢复[33]。③IL-1、TNF-α可抑制EPO的产生，高迁移率族蛋白B1（HMGB1）则可以促使EPO信号传导障碍[34]–[35]，阻碍红系祖细胞向成熟红细胞增殖分化。④大量炎症因子导致异常激活的巨噬细胞可直接对血细胞产生破坏[36]–[38]等。

4. 其他：另有研究[7]观察到血小板计数与CD56阳性细胞计数之间有很强的相关性。NK细胞（表达CD56）是骨髓功能恢复过程中最早出现的造血细胞之一[39]–[40]，血小板计数和NK细胞数量之间的关联可能反映骨髓功能。CAR-T细胞治疗1个月时血细胞计数完全恢复的患者巨噬细胞来源趋化因子（MDC）和生长因子（包括血管内皮生长因子、转化生长因子和成纤维细胞生长因子）的水平较高[12]，虽然小样本研究显示其与血细胞计数恢复之间无统计学意义的相关性，但仍能表明其对促进造血功能恢复可能起一定作用，骨髓微环境的变化可能有助于造血祖细胞的恢复。

三、CAR-T细胞治疗相关长期血细胞减少的处理对策

（一）早期风险分层

Rejeski等[22]进行了一项真实世界的多中心回顾性观察性研究，整合了258例接受axi-cel或tisa-cel治疗的复发/难治大B细胞淋巴瘤患者基线临床数据，定义了三种中性粒细胞的恢复模式：①快速恢复：中性粒细胞恢复至正常水平（>1.5×10^9^/L）且不再下降至<1.0×10^9^/L；②间歇性恢复：中性粒细胞短暂恢复（>1.5×10^9^/L）,在21 d后再次下降至<1.0×10^9^/L；③再生障碍型：重度中性粒细胞下降（<0.5×10^9^/L）持续≥14 d。

据此开发了CAR-HEMATOTOX模型[22]，其中包括基线血细胞计数（ANC、血小板、血红蛋白）及炎症标志物（CRP和铁蛋白）（表1），根据表中内容进行积分，用于预测CAR-T输注后发生长期血细胞减少的风险及预后。CAR-HEMATOTOX评分0～1 分的患者血细胞恢复模式更多表现为快速恢复型；而评分高（≥2 分）的患者则更多表现为再生障碍型模式，容易发生长期血细胞减少，且程度更重、持续时间更长，患者无进展生存期及总生存期更短。

**表1:** CAR-HEMATOTOX（HT）模型评分标准[22]

评分（分）	基线特征				
	PLT（×10^9^/L）	ANC（×10^9^/L）	HGB（g/L）	CRP（mg/L）	铁蛋白（µg/L）
0	>175	>1.2	>90	<30	<650
1	75～175	<1.2	>90	>30	650～2 000
2	<75				>2 000

注 总分：0～5分；ANC：中性粒细胞绝对计数；CRP：C反应蛋白

根据CAR-HEMATOTOX模型，将0～1 分定义为低风险，≥2 分定义为高风险。在输注CAR-T细胞前，可早期评估风险分层，对于高风险患者应加强防护，指导患者注意环境及个人卫生，必要时可行保护性隔离；并可预防性给予抗细菌、真菌治疗及集落刺激因子支持等治疗，防止潜在感染并发症的发生。

（二）处理对策

在完成早期风险分层后，监测血常规及血清炎症标志物变化、预防/控制感染以及适当的支持治疗至关重要。同时，CRS及ICANS等级作为长期血液学毒性的影响因素，应依据相关指南[41]加强管理。

对于中性粒细胞减少症患者，可予保护性隔离。+14 d起或当ANC<0.5×10^9^/L时，可以给予G-CSF，用于缩短中性粒细胞减少的持续时间。对于已有发热或感染风险高（高危因素[42]包括ALL、既往30 d内感染病史、难治性疾病、回输前粒细胞缺乏、既往接受过多线抗肿瘤方案以及发生≥3级CRS，IL-6出现双峰可预测严重感染）的患者可更早给予。目前已有报道早期（+5 d）预防性给予G-CSF不会增加严重CRS/ICANS发生的风险，且对CAR-T细胞的增殖、抗肿瘤效应及预后均无明显影响[43]。GM-CSF是促进细胞因子风暴发生的关键蛋白，刺激单核细胞生成并产生IL-6、IL-10，存在使CRS加重的风险[44]–[45]，建议治疗全程禁止使用GM-CSF。

对于贫血和血小板减少症患者，当HGB<60 g/L或观察到明显的贫血症状时，应及时输注红细胞或给予EPO[21]；当血小板低于20×10^9^/L或观察到出血症状时，可进行血小板输注或给予TPO、TPO受体激动剂等[21],[46]治疗；同时监测凝血功能，当纤维蛋白原<1 g/L时，可给予纤维蛋白原浓缩物或冷沉淀替代治疗[47]–[48]。

目前，TPO受体激动剂已经成功用于治疗免疫性血小板减少性紫癜、再生障碍性贫血以及HSCT后引起的血小板减少症[49]–[51]。一项单中心治疗经验[52]，对93例DLBCL成年患者（中位年龄为69岁）输注CD19 CAR-T细胞后21～28 d进行了血细胞计数检查。在出现严重长期血细胞减少的6例患者（6.5％，4例接受tisa-cel治疗；2例接受axic-cel治疗）中，给予TPO受体激动剂治疗（4例给予艾曲波帕，1例给予罗米洛司汀，1例给予以上两种药物），同时提供输血、G-CSF及其他支持治疗。所有患者的血小板计数在TPO 受体激动剂输注后均>20×10^9^/L，中位恢复时间27（6～38）d。虽然已有关于TPO激动剂治疗CAR-T相关性长期血小板减少的报道，但各中心对其使用尚未形成普遍共识及标准化，仍需进一步完善系统治疗方案。一般而言，对于持续超过30 d的血细胞计数减少，建议骨髓穿刺和（或）组织检查，以评估原发肿瘤的状况，及时排除HLH和骨髓增生异常综合征等继发性恶性肿瘤[53]。如果骨髓活检诊断为再生障碍性贫血，且没有疾病复发及继发性恶性肿瘤的证据，患者可以考虑启用TPO受体激动剂治疗。

大多数患者的血细胞计数通过成分输血及输注刺激因子后可得到恢复。对于一般治疗后仍难以纠正的患者，可考虑在接受CAR-T细胞治疗后早期进行HSCT，促进骨髓造血系统重建[54]–[57]。在一项研究[58]中报告了不同疾病的患者在输注CD19 CAR-T（共12例，9例接受tisa-cel治疗，2例接受axic-cel治疗，1例接受brexu-cel治疗）后接受HSCT（共12例，9例自体HSCT，3例异基因HSCT）治疗持续性中性粒细胞减少症的结果。CAR-T输注后中位时间69（35～617）d给予HSCT，中性粒细胞缓解率为82％，所有患者HSCT后中性粒细胞恢复的中位时间为15（6～124）d。此外，有研究报道[59]–[61]CAR-T细胞治疗达到微小/可测量残留病或分子残留病灶阴性的完全缓解后接受allo-HSCT的白血病患者比单独接受CAR-T细胞治疗的患者有更长的无病生存期。对于高风险患者，预防性采集自体干细胞已被提出作为缓解长期血细胞减少的一种策略[62]–[63]。对于已发生长期血细胞减少的患者，进行自体或异基因HSCT的最佳时间仍然需要进一步研究。

四、总结

CAR-T细胞治疗后长期血细胞减少的病理生理学仍然知之甚少，可能与CAR-T结构、既往治疗、桥接治疗、基线骨髓内肿瘤负荷、基线血细胞计数、ICANS/CRS、炎症标志物/细胞因子水平等因素相关。

治疗前评估相关风险，可回顾以往治疗过程中造血细胞下降的强度和持续时长、G-CSF的使用情况等，并根据CAR-HEMATOTOX模型对患者进行长期血细胞减少风险预测。识别高风险患者，可提前采集自体干细胞备用，选择合适的CAR-T产品及清淋巴细胞预处理方案，提供更严密的护理支持，预防性给予G-CSF及经验性广谱抗生素治疗，或符合移植条件的患者可联合CAR-T及HSCT治疗缓解不良反应，同时也要根据相关指南及时预防及处理CRS、ICANS、感染等其他并发症的发生。

总之，有关CAR-T细胞治疗后长期血细胞减少的具体发生机制尚不明确，大部分的研究都是单中心的，小样本量的结果在某些影响因素方面尚存在争议。还需要进行更多大样本量、多中心的研究，形成更加系统、明确的认识。
